# Development of the Nervous System of *Carinina ochracea* (Palaeonemer-tea, Nemertea)

**DOI:** 10.1371/journal.pone.0165649

**Published:** 2016-10-28

**Authors:** Jörn von Döhren

**Affiliations:** Institute of Evolutionary Biology and Ecology, University of Bonn, Bonn, Germany; Universitat Wursburg, GERMANY

## Abstract

The various clades of Lophotrochozoa possess highly disparate adult morphologies. Most of them, including Nemertea (ribbon worms), are postulated to develop via a pelagic larva of the trochophora type, which is regarded as plesiomorphic in Lophotrochozoa. With respect to the nervous system, the trochophora larva displays a set of stereotypic features, including an apical organ and trochal neurites, both of which are lost at the onset of metamorphosis. In the investigated larvae of Nemertea, the nervous system is somewhat divergent from the postulated hypothetical trochophore-like pattern. Moreover, no detailed data is available for the “hidden” trochophore larva, the hypothetical ancestral larval type of palaeonemertean species. Therefore, the development of the nervous system in the larva of *Carinina ochracea*, a basally branching palaeonemertean species, was studied by means of immunofluorescence and confocal laserscanning microscopy. Like in the other investigated nemertean larvae, the prospective adult central nervous system in *C*. *ochracea* develops in an anterior to posterior direction, as an anterior brain with paired longitudinal nerve cords. Thus, development of the adult nervous system in Nemertea is largely congruent with currently accepted hypotheses of nervous system development in Spiralia. In early development, transitory apical, serotonin-like immunoreactive flask-shaped cells are initially present, but the trochal neurites that have been considered as pivotal to lophotrochozoan development, are absent. In the light of the above stated hypothesis, trochal neurites have to be interpreted as reduced in Nemertea. On the other hand, due to the unsettled systematic status of Palaeonemertea, more comparative data are desirable to answer the remaining questions regarding the evolution of nervous system development in Nemertea.

## Introduction

Lophotrochozoa (in the strict sense of e.g. [1]) is characterized by highly disparate adult morphologies. This phenomenon, recently termed “lophotrochozoan bodyplan paradox,” [2] has hampered comparative investigations on the adults of the various clades. The solution offered was comparing developmental stages instead of the very dissimilar adult morphologies [2–5]. To elucidate the comparative development of the nervous system, fluorescent labeling of serotonin-like (5HT-lir), FMRFamide-like (RFa-lir), and acetylated α-tubulin-like (tub-lir) immunoreactivity, and its detection by confocal laser scanning microscopy (IF/CLSM), has recently been performed on a broad systematic scale [5,6]. Thus, a hypothesis on the ancestral nervous system architecture in Lophotrochozoa is currently emerging [2,7]. Nevertheless, Nemertea (ribbon worms) have to be considered underrepresented in this respect.

Nemertea comprises approximately 1300 unsegmented, worm-shaped lophotrochozoan species [8,9]. They are characterized by an endothelialized blood vascular system, an eversible proboscis housed in a fluid-filled secondary body cavity (the rhynchocoel), and a ring-shaped brain, encircling the proboscis insertion instead of the mouth opening [10,11]. Currenly available immunohistochemical observations on nervous system development in Nemertea draw a heterogeneous and still fragmentary picture [12,13]. Detailed information on the formation and architecture of the larval nervous system, based on IF/CLSM, is only available in members of Neonemertea [14–18]. Neonemertea is a monophyletic taxon [19–21] in which arguably derived larval types occur, of which the pilidium might be the most well-known [13,22,23]. Instead, the so-called “hidden” trochophore larva of the more basally branching palaeonemertean genus *Carinoma* has been hypothesized to represent the ancestral larval type in Nemertea [22,24]. Detailed data on the development of the nervous system in palaeonemertean species, however, are virtually non-existent.

To contribute to filling the existing informational gaps, the development of the nervous system was studied in larval stages of the palaeonemertean species *Carinina ochracea*. Data on 5HT-lir, RFa-lir, and tub-lir were obtained by means of IF/CLSM. Furthermore, synapsin-like immunoreactivity (syn-lir) [25] was assessed for its suitability to label neuropil components of the nervous system in nemerteans. A species of *Carinina* was chosen as subject for the investigation, since species of this genus arguably possess the most ancestral position of the central nerous system (CNS) in Nemertea: In adult animals the CNS is located at the base of the epidermis, distal to its basal extracellular matrix [26,27]. The ancestral character of the adult nervous system raises the expectation that the nervous system will show ancestral trochozoan-like features during its development. The investigation represents an initial contribution to a sequence of detailed and comparative investigations on the development of the neuromuscular system in a broad range of nemertean species.

## Materials and Methods

### Species and collection site

Sexually mature females and males of *Carinina ochracea* Sundberg, Chernyshev, Kajihara, Kånneby and Strand, 2009 (Palaeonemertea, Nemertea) were obtained by digging in a sandflat near Pouldohan (Dpt. Finistère, France; 47°50'N 3°53'W) at low-tide between May and August of the years 2011 to 2013 during visits at the Station de Biologie Marine de Concarneau (Dpt. Finistère, France). It is confirmed that neither *C*. *ochracea* is protected, nor is the access to the collecting site in any way legally restricted. Animals were collected with oral permission of the local marine biological station (Station de Biologie Marine de Concarneau). Written permissions were not required. For confirmation of species identification, DNA Extraction, PCR, nucleotide sequencing, and BLAST of COI “barcode-region” [28] were performed (S1 Appendix). Animals were brought to the Institute of Evolutionary Biology and Ecology of the University of Bonn (Germany), where they were kept at 18°C in plastic boxes with lid (approx. 1.5 liter capacity) filled with sand from the collecting site and artificial sea water. Water was changed once per week. For research on unprotected invertebrate animals, no approval is required by the University of Bonn.

### Obtaining gametes and larval culture

Oocytes and sperm were obtained at 18°C by dissecting the gonad region of females and males with aid of a stereomicroscope and fine forceps and a razor blade in ultra-filtered (0.2 μm) natural sea water (NSW) from the collecting site. Only the oocytes retained by a 100-μm mesh were kept for fertilization. Oocytes were kept in glass bowls (approx. 200-ml capacity) filled with approx. 100 ml of ultra-filtered NSW from the collecting site for approximately 30 min prior to fertilization. For fertilization several (6–10) drops of sperm suspension diluted in ultra-filtered NSW from the collecting site were added to the oocytes. During the course of the study several males and females were used to obtain numerous batches of larvae. Developing embryos/larvae were kept at 18°C at a light-dark cycle of 16:8 hours. During the first 2 days of development, water was changed daily with ultra-filtered NSW from the collecting site. Afterwards, the larvae were transferred to plastic boxes with a lid (approx. 1.5 liter capacity) filled with 1 liter of a mixture of equal parts of artificial sea water and natural sea water from the collecting site filtered through a 20-μm mesh. Three times a week, half of the total volume (500 ml) was filtered through a 50-μm mesh by reverse filtering. Water changes were done with the sea water mixture mentioned above. Although several food items (including *Tetraselmis subcordiformis* and *Saccharomyces cerevisiae*) were offered, the larvae ceased to develop further after the 10^th^ day and disappeared from the culture after approximately 3 weeks.

### Sampling and fixation of larval stages

Sampling and fixation of larvae started after 24 hours of development, at the onset of swimming of the larvae, to avoid selection of malformed developmental stages. Only normal looking, rounded specimens were chosen. Subsequently, larvae were fixed at 1, 2, 3, 4, 5, 7, and 10 days post-fertilization (*dpf*). Prior to fixation, larvae were washed with 3 changes of ultra-filtered artificial sea water. From the 3^rd^ day of development onward, larvae were anesthetized with ultra-filtered (0.2 μm) 3.5% MgCl_2_ (prepared from equal amounts of 7% MgCl_2_ (VWR Chemicals) solution in distilled water and artificial sea water) at room temperature for 15 min. Subsequently, larvae were fixed at 18–20°C in ultra-filtered (0.2 μm), 4% para-formaldehyde solution (PFA, freshly prepared from one part of a 16% PFA solution (Electron Microscope Science) and three parts of artificial sea water) for 30 min in glass bowls (approx. 4 ml capacity) coated with Sigmacote (Sigma-Aldrich) to avoid extensive sticking of the larvae to inside of the bowls. To stop the fixation process, larvae were washed in 3 changes of 0.1M phosphate buffer saline (PBS) at pH 7.4 to which 0.01% NaN_3_ (Carl Roth) was added for 10 min each. Larvae were stored in the same buffer in siliconized polypropylene microcentrifuge tubes (1.7 ml capacity) (Sigma-Aldrich).

### Immunohistochemistry, fluorescent labeling, and mounting

Larvae were washed in 3 changes of PBT (PBS with 0.3% Triton X-100 (Sigma-Aldrich) added) for 10 min each prior to blocking in PBT with 10% normal goat serum (NGS, Sigma-Aldrich) for 2 hours at room-temperature. Subsequently, the larvae were incubated with the primary antibodies diluted in PBT at 18°C on a rocking table overnight. Primary antibodies applied were against serotonin (5’-HT) produced in rabbit (20080, Immunostar, polyclonal) at a dilution of 1:1000, FMRFamide produced in rabbit (ab10352-50, abcam, polyclonal) at a dilution of 1:1000, acetylated α-tubulin produced in mouse (T7451, Sigma-Aldrich, monoclonal) at a dilution of 1:300, and synapsin produced in mouse (anti SYNORF1 = 3C11, monoclonal; Developmental Studies Hybridoma Bank, DSHB, University of Iowa) at a dilution of 1:10, each from a stock solution of 1mg/ml. The antibody 3C11 (anti SYNORF1) was deposited to the DSHB by E. Buchner (DSHB Hybridoma Product 3C11 (anti SYNORF1)) [25]. For double-staining the antibodies were appropriately combined: primary antibody produced in rabbit (i.e. anti serotonin or anti FMRFamide) with primary antibody produced in mouse (i.e. anti acetylated α-tubulin or anti SYNORF). To evaluate potential cross reactions of the primary antibodies, different combinations of the antibodies were tested. Additionally, single-staining was performed with each primary antibody. The primary antibodies were removed by three quick washes followed by three washes of 15 min each in PBT. Respective secondary antibodies were subsequently applied in PBT: goat anti rabbit conjugated to Alexa Fluor 488 (A11008, ThermoFisher, polyclonal) at a dilution of 1:100 and goat anti mouse conjugated to either Alexa Fluor 568 (A11004, ThermoFisher, polyclonal) at a dilution of 1:150 (against SYNORF1), or Alexa Fluor 633 (A21052, ThermoFisher, polyclonal) at a dilution of 1:100 (against acetylated α-tubulin) each from a stock solution of 1 mg/ml. To assess for unspecific binding of the secondary antibodies, controls were run by omitting the primary antibody from the antibody staining procedure outlined above. The secondary antibodies were washed out using the same procedure as outlined for primary antibodies. After the final wash the larvae were fastened to cover glasses (#1, Thermo Scientific) coated with 0.01% poly-L-lysine-hydrobromide (Sigma-Aldrich). The preparations were dehydrated using a graded series of isopropanol (70%, 85%, 95%, twice 100%) for 1 min each followed by 3 changes of Murray’s Clear (= BABB: 1 part of benzyl alcohol (Carl Roth) mixed with 2 parts of benzyl benzoate (Sigma-Aldrich)) for 10 min each. The preparations were then mounted in Murray’s Clear using small pieces of modeling clay as spacers between glass slide and cover glass. The gaps between glass slide and cover glass were sealed with nail polish. Before they were examined, the larvae were allowed to clear over night at 4°C.

### Signal detection and image processing

To follow the development of the nervous system, a minimum of six larvae per stage were examined. For signal detection a Leica TCS/SPE confocal laser scanning system mounted on a Leica DM 2500 microscope was used. Alexa Fluor 488 was excited with the 488 nm (light blue), Alexa Fluor 568 with the 532 nm (green), and Alexa Fluor 633 with the 635 nm (red) laser lines at intensities indicated in S1 Table. All images were recorded with a 40x dry objective with a numerical aperture (NA) of 0.75 with the pinhole diameter set to one airy disc size. For double-stained specimens the sequential excitation/detection setting was used. Stacks of images were recorded with a virtual thickness of 0.92 μm (*1-dpf*) or 0.88 μm (all remaining specimens) at 1024×1024 pixels at 8-bit image depth except for *3-dpf* anti-acetylated α-tubulin, which was recorded at 12-bit image depth. Details on the variable settings (i.e. laser intensity, gain, offset, and recorded emission spectra) are listed in S1 Table. In order to minimize the unspecific signals of the epidermal mucus gland cells (see below) stacks were subjected to the image processing protocol as outlined in S2 Appendix. Details on image processing are listed in S1 Table. Z-projections were produced with the Z-Project command in ImageJ version 1.50e (Wayne Rasband, NIH) of the Fiji distribution package [29,30]. In the image showing anti-actylated α-tubulin staining, only the optical sections in the middle of the stack were projected. Additionally, the maximum gray value was set to 37% of the maximum value in order to equilibrate the strong ciliary signal with the weaker neurite signals. The projections were gamma corrected with the gamma command in ImageJ version 1.50e of the Fiji distribution package (Wayne Rasband, NIH) [29,30]. The effect of image processing is illustrated in S1 Fig. For additional reference, all stacks before image enhancement have been uploaded to MorphDBase (www.morphdbase.de) (S1 Table). Image plates and line drawings were produced with Adobe Illustrator CS6 (Adobe Systems Incorporated).

## Results

### Immunoreactivity in *Carinina ochracea*

**Fig 1:**
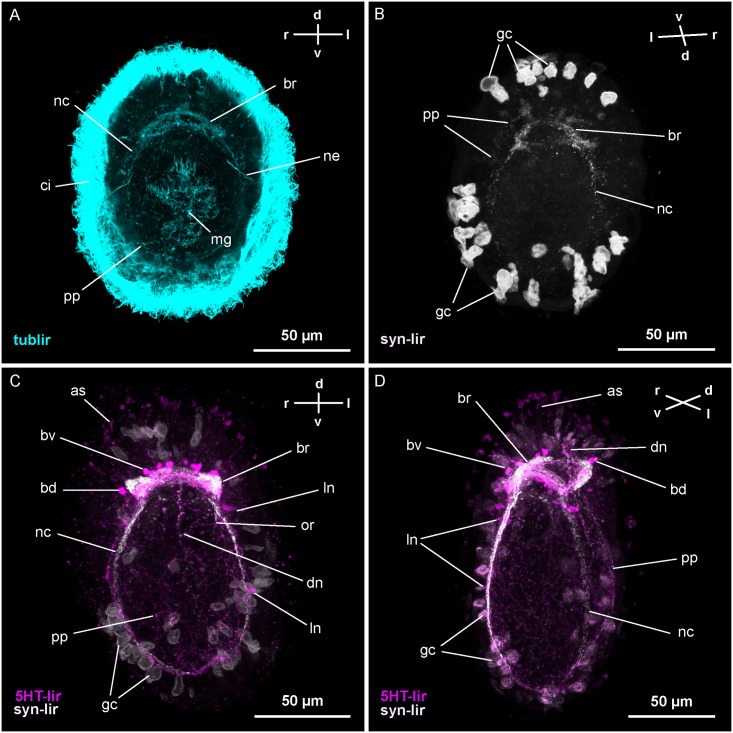
Z-Projections of immuno-stained specimens of *Carinina ochracea*. Orientation indicated by compass (d, dorsal; l, left; r, right; v, ventral); apical is up. A and B: larva three days post-fertilization (*3-dpf*). A: Acetylated α-tubulin-like immunoreactivity (tub-lir) (γ: 0.89). Tub-lir has the strongest signals in the epidermal cilia (*ci*), the cilia of the midgut (*mg*) and the nephridial tubules (*ne*). Neuronal tub-lir signals outline the brain-ring (*br*), the developing lateral nerve cords (*nc*) and the peripheral plexus (*pp*). B: Synapsin-like immunoreactivity (syn-lir) (γ: 0.82). Syn-lir marks the developing neuropil of the brain-ring (*br*), especially the ventral part and the lateral nerve cords (*nc*). Additionally, the peripheral plexus (*pp*) displays syn-lir. Note the strong unspecific signals of large epidermal mucus gland cells (*gc*) partly extruded at fixation. C and D: Combined syn-lir and serotonin-like immunoreactivity (5HT-lir). C: Larva at *5-dpf* (γ: 0.89 (5HT-lir), γ: 0.88 (syn-lir)). Syn-lir is visible in the brain-ring neuropil (*br*) the lateral nerve cords (*nc*), the peripheral plexus (*pp*), and the dorsal nerve (*dn*). Additionally, the first signals of the oral ring neurite bundle (*or*) are detectable. Note the unspecific signals of epidermal mucus gland cells (*gc*). The 5HT-lir neurons associated with the ventral (*bv*) and dorsal (*bd*) brain-ring (*br*) are distinct, as are the 5HT-lir neurites of the brain-ring. The peripheral plexus (*pp*) and the apical signals (*as*) are displaying 5HT-lir. Along the 5HT-lir neurite signals of the lateral nerve cords (*nc*) the first paired associated neurons (*ln*) can be seen. A weak dorso-median 5HT-lir signal indicates the neurites of the dorsal nerve (*dn*). D: Larva at *10-dpf* (γ: 0.89 (5HT-lir), γ: 0.88 (syn-lir)). Syn-lir is still weaker in the dorsal-most part of the brain-ring neuropil (*br*) compared to the ventral part and the lateral nerve cords (*nc*). The peripheral plexus (*pp*) and the anteriorly elongated dorsal nerve (*dn*) display syn-lir. The oral ring neurite bundle (*or*) has become more prominent and paired pre-oral neurite bundles (*po*), connecting to the ventral portion of the brain-ring, are visible. Note the unspecific signals of epidermal mucus gland cells (*gc*) and the weaker signal of the left lateral nerve cord (*nc*) due to signal attenuation. The 5HT-lir neurons associated with the brain-ring (*bv* and *bd*) and the lateral nerve cords (*ln*) have increased in numbers. The 5HT-lir dorsal nerve signal (*dn*) extends anteriorly beyond the brain-ring neurites. Apical 5HT-lir signals (*as*) are numerous but comparably weak.

Since development is not synchronous within a batch of larvae, a gradient of development could be identified at each stage. Only signals that were consistent in several larvae are documented (Table 1). In many larvae, bilaterally occurring neural structures seem to display immunoreactivity slightly earlier on one side of the body than on the other. Cross reactivity of antibodies was not detected in any combination tested. Thus, the different combinations were used to relate those signals to each other, of which the respective antibodies could not be double-stained. Antibodies against acetylated α-tubulin reliably mark microtubules present in the neurites, but neuronal perikarya are not consistently labeled. Therefore, it is impossible to reliably estimate the total number of neurons. Furthermore, in *Carinina ochracea* the ciliary signals are generally stronger than the signals produced by the microtubules of the neurites, thus obscuring the signals of the latter (Fig 1A). A similar problem with antibodies against β-tubulin and tyronisated α-tubulin negate their use as an alternative. Although not double-stained with acetylated α-tubulin antibodies, antibodies against synapsin (SYNORF1) show signals largely congruent to tub-lir in larvae of *C*. *ochracea* (Fig 1B). Therefore, a combination of tub-lir and syn-lir seems most suitable for assessing the overall architecture of the nervous system. The signals obtained in *C*. *ochracea* with the employed antibodies against neuroactive substances, i.e., 5HT-lir and RFa-lir, show mostly signals that are morphologically correspondent to neurites and neuronal perikarya (but see below for RFa-lir). Along some of the neurites the antibodies also mark club-shaped extensions of smaller size than typical perikarya. These extensions are provisionally identified as growth cones of developing neurites.

**Table 1:** Development of nervous system specific immunoreactivity in larvae of *Carinina ochracea*.

age of larva	acetylated α-tubulin-like immunoreactivity	synapsin-like immunoreactivity	serotonin-like immunoreactivity	FMRFamide-like immunoreactivity
***1-dpf***	median caudal neuron	no signals	2 apical neurons, 2 caudal neurons, lateral longitudinal neurites	no signals
***2-dpf***	medio-dorsal caudal neuron, peripheral plexus neurites	no signals	1st brain ring neurites, 1st pair of ventral brain neurons, dorsal longitudinal neurite; disappeared median apical neuron	dorso-median neurite mass
***3-dpf***	brain ring, nerve cords; disappeared caudal neurons	brain ring, nerve cords, peripheral plexus	peripheral plexus, 2 pairs of ventral and 2 pairs of dorsal brain neurons; disappeared medio-ventral apical neuron, caudal neurons, lateral and dorsal longitudinal neurites	brain ring neurites, 1 pair of dorsal brain ring neurons, nerve cord neurites
***4-dpf***	anal commissure, pre-oral neurites, mouth ring neurites	no additional signals	3 pairs of ventral and 3 pairs of dorsal brain neurons, nerve cord neurites, 1 pair of nerve cord neurons	3 pairs of dorsal brain ring neurons (1 anterior, 2 posterior of brain ring neurites), pre-oral neurites, mouth ring neurites
***5-dpf***	“dorsal nerve” neurite bundle	“dorsal nerve” neuropil, mouth ring neuropil	5 pairs of ventral brain neurons, 2 pairs of nerve cord neurons, “dorsal nerve” neurites	5 pairs of dorsal brain ring neurons (2 anterior, 3 posterior of brain ring neurites), paired suboral neurites with 1 dorso-lateral neuron each
***7-dpf***	no additional signals	pre-oral neuropil	6 pairs of ventral brain neurons, 5 pairs of nerve cord neurons (3 anterior, 2 posterior of mouth opening)	6 pairs of dorsal brain ring neurons (2 anterior, 4 posterior of brain ring neurites), paired ventro-lateral neurons with neurites connected to suboral neurites
***10-dpf***	no additional signals	no additional signals	7–8 pairs of ventral, 4 pairs of dorsal brain ring neurons	9 pairs of dorsal brain ring neurons (4 anterior, 5 posterior of brain ring neurites), 2nd pair of dorso-lateral neurons with neurites connected to suboral neurites

*dpf*, days post-fertilization

The antibody against FMRFamide suffers from a higher unspecific background staining than the other antibodies employed. Therefore, identification of RFa-lir perikarya remains somewhat ambiguous. The exact numbers of neuronal signals could thus not be determined. In the case of the neurons of the dorsal portion of the brain and of the lateral nerve cords a few RFa-lir neuronal signals are seen to be in contact with the epidermis in some specimens, but not in others that seem more advanced. These signals can be addressed as developing RFa-lir neurons migrating inwards from the dorso-lateral and lateral epidermis respectively. In contrast, several cells of the midgut epithelium show RFa-lir, but these signals are not consistent among different larvae. In the present study it could not be substantiated whether these cells show increased background staining due to different cell morphologies or whether their signal is specific RFa-lir.

A general problem to all primary antibodies used, is the presence of large, epidermal mucus gland cells: Excess secondary antibodies tend to bind to them, producing comparably strong but unspecific signals. Since synapsin-like material seems to be relatively scarce in the larva of *C*. *ochracea* in younger stages, syn-lir staining seems to be especially prone to this type of overstaining (Fig 1B–1D).

### Development of acetylated α-tubulin-like immunoreactive neural elements

Prior to the onset of the development of the major components of the central nervous system (CNS, i.e. brain-ring and paired lateral nerve cords) at *3-dpf*, tub-lir is indistinct and only detected in the minority of larvae examined. At *1-dpf* tub-lir is displayed in a medially located, caudal neuron but only in a few of the examined specimens. The neuron is flask-shaped with one stout process in connection with the larval surface. In a few specimens an apical neuron of similar appearance also displays tub-lir. By *2-dpf* a second neuron on the caudal pole, dorsal of the first, is visible. Its morphology is similar to the other caudal neuron. In some preparations an apical flask-shaped neuron ventral of the apical pit also displays tub-lir. It is likely identical with the apical neuron detected at *1-dpf*. Irregularly distributed tub-lir is detectable forming a network of neurite signals throughout the body. However, due to the obscuring signal of developing cilia in the adjacent epidermal cells, the exact arrangement of the peripheral plexus remains elusive. In a few specimens two tub-lir signals of the two caudal neurons are still visible at *3-dpf*. No unambiguously neuronal tub-lir signals are observed at the anterior end of the larva at this stage. In the anterior third of the larvae the developing brain-ring neurites start showing tub-lir. The first neurites of the developing lateral nerve cords also display tub-lir (Fig 1A). They are connected ventro-laterally to the brain-ring and extend posteriorly to the level of the mouth opening. Tub-lir is also detectable in the peripheral plexus distal of the signals of the developing CNS (Fig 1A). During further development tub-lir of the nervous system becomes successively more obscured by the strong microtubule signals in developing cells of the epidermis and midgut epithelium. The posterior connection of the nerve cords, the anal commissure, is faintly seen in the larvae at *4-* to *5-dpf*. Around the same time, a ring-shaped bundle of neurites around the mouth opening (oral ring neurite bundle) and paired bilateral neurite bundles, connecting the frontal arc of the oral ring neurite bundle with the ventral brain-ring (pre-oral neurite bundles), display tub-lir. A dorso-median, longitudinal, tub-lir neurite bundle, representing the so-called “dorsal nerve,” can barely be made out against the background of epidermal tub-lir signals in advanced specimens at *5-dpf*.

### Development of synapsin-like immunoreactive neural elements

In *C*. *ochracea*, syn-lir is first detectable in *3-dpf* larvae. It is observed in the brain-ring and in the lateral nerve cords that start to extend posteriorly from the ventral portion of the brain-ring (Fig 1B). In the brain-ring, syn-lir is generally stronger in the ventro-lateral part of the brain-ring close to the transition to the lateral nerve cords. It is most indistinct in the dorsal-most, median part of the brain-ring, where it comprises more loosely arranged, isolated signals. During subsequent development the syn-lir signals of the brain-ring and nerve cords become more prominent. The latter also keep elongating towards the posterior pole of the larva (Fig 1C). Until the most advanced stages studied, the syn-lir signal of the brain-ring remains most indistinct in its dorso-median part (Fig 1D). The peripheral plexus also shows distinct but irregular syn-lir in larvae at *3-dpf* (Fig 1B). During further development the syn-lir signals in the peripheral plexus become more prominent and are still detected in the most advanced stages studied (Fig 1D). From *5-dpf* onward, syn-lir signals are additionally seen in the dorsal nerve, which subsequently elongates; first posteriorly and later also anteriorly beyond the brain-ring (Fig 1C and 1D). By *5-dpf*, the neuropil of the oral ring neurite bundle is seen by its first bilaterally symmetric, elongated syn-lir signals posterior of the brain-ring, between the lateral nerve cord signals (Fig 1C). Later, the signal forms an incomplete ring that is open posteriorly. It is not until about *7-dpf* that the syn-signal of the oral ring neurite bundle is completely closed. In the pre-oral neurite bundles, syn-lir signals are not apparent until *7-dpf*. In the anal commissure, syn-lir was not observed in any of the stages examined (Fig 1D).

### Development of serotonin-like immunoreactive neural elements

**Fig 2:**
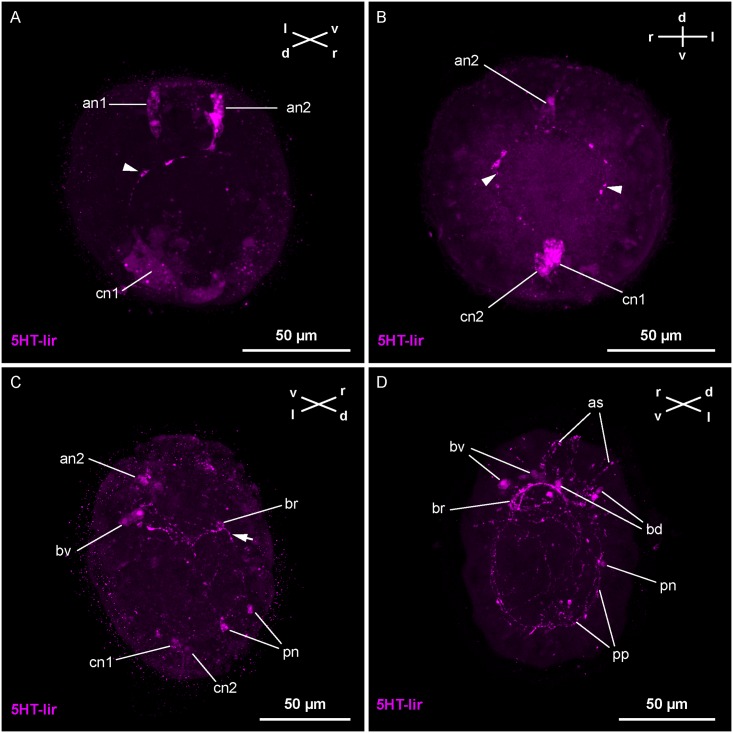
Z-Projections of immuno-stained specimens of *Carinina ochracea*. Serotonin-like immunoreactivity (*5HT-lir*). A: gastrula stage one day post-fertilization (*1-dpf*) (γ: 0.84). Two apical neurons (apical median: *an1*, medio-ventral: *an2*) and one caudal neuron (*cn1*) display 5HT-lir. Note the neurite connecting *cn1* and *an2* (arrowhead). B: Post-gastrula stage larva at *1-dpf* (γ: 0.77). Apically, only the medio-ventral neuron (*an2*) is detectable. Caudally a second, more dorsally located neuron displays 5HT-lir (*cn2*). A pair of longitudinal 5HT-lir neurite-like signals connects *cn1* and *an2* (arrowheads). C: Larva at *2-dpf* (γ: 0.9). The first-formed 5HT-lir neurons show weak and patchy signals. Posterior of *an2*, the 5HT-lir brain-ring neurites (*br*) are seen, along with ventrally located neurons displaying 5HT-lir (*bv*). The dorso-median neurite signal is only visible in its anterior extension (arrow). Note the paired dorsally located 5HT-lir neurons (*pn*), possibly contributing to the forming peripheral plexus. D: Larva at *3-dpf* (γ: 0.9). The first-formed signals have ceased to display 5HT-lir. Dorsally and ventrally located 5HT-lir neurons (*bd*, *bv*) are visible associated with the 5HT-lir brain-ring neurites (*br*). A 5HT-lir peripheral plexus (*pp*) has formed in the vicinity of the peripheral neurons (*pn*). At the apical pole of the larva, several weak 5HT-lir signals (*as*) are present.

**Fig 3:**
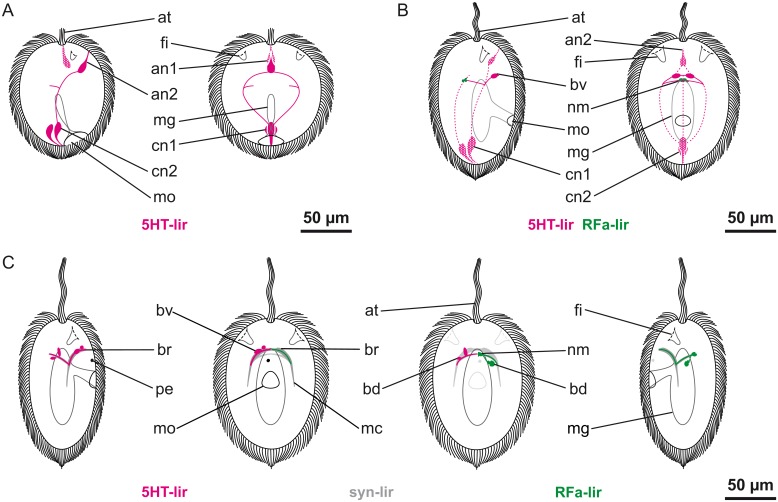
Schematic representation of nervous system development of *Carinina ochracea*. Serotonin-like (*5HT-lir*) and FMRFamide-like (*RFa-lir*) immunoreactivity are color coded in magenta and green respectively; the general nervous system architecture as outlined by synapsin-like immunoreactivity (*syn-lir*) is shown in grey. All larval stages are drawn to scale with apical tuft (*at*), epidermal cilia, fronto-lateral epidermal invaginations (*fi*), midgut (*mg*), mouth opening (*mo*), and the pigmented larval eye (*pe*) serving as landmarks (cilia of the frontal epidermal invaginations, the stomodaeum, the midgut, and epidermal cilia in the aspect of view omitted), apical is up. A and B: left: lateral view from right, right: ventral view. Transitory neuronal/neuritic elements are indicated by stippling/dotted lines. A: Post-gastrula stage larva at one day post-fertilization (*1-dpf*). B: Larva at *2-dpf*. C: Larva at *3-dpf*. From left to right: lateral view from right, ventral view, dorsal view, lateral view from left. Since the nervous system is roughly symmetrical, only one side is shown (left: 5HT-lir, right: RFa-lir). For clarity, the peripheral plexus is not shown. Further explanations, see text.–*bd*, dorsal brain neuron; *br*, brain-ring; *bv*, vental brain neuron; *an1*, 1^st^ apical median (transitory) neuron; *an2*, 2^nd^ apical medio-ventral (transitory) neuron; *nc*, lateral nerve cord; *cn1*, 1^st^ caudal median (transitory) neuron; *cn2*, 2^nd^ caudal medio-dorsal (transitory) neuron; *nm*, dorso-median neurite mass.

The first 5HT-lir signals are detected in the embryo in the gastrula stage, which was observed in some batches of larvae at *1-dpf* (Fig 2A). The signals comprise two elongated cells at the animal pole of the gastrula, one in an apical, median and the other in a more medio-ventral position. On the vegetal pole, a large cell that borders the blastopore in a dorsal position also shows 5HT-lir (Fig 2A). In some specimens, a neurite-like 5HT-lir signal is visible connecting the caudal with the more ventrally located apical neuron (Fig 2A). In more advanced post-gastrula stages at *1-dpf*, the two apical neurons and the caudal neuron have changed shape to a flask-like outline with a stout, neck-like process projecting to the epidermal surface (Figs 2B and 3A). The more ventrally located apical neuron and the caudal neuron are connected by bilaterally located 5HT-lir neurite signals. The neurites extend longitudinally on both sides of the developing midgut (Figs 2B and 3A). Just anterior of the midgut each of the signals give of a short branch that is oriented dorsally (Fig 3A). A second caudal 5HT-lir flask-shaped cell is detectable. It is situated dorsal of the first-formed caudal neuron. Like the other early 5HT-lir neurons, it possesses a stout process that is reaching to the epidermal surface (Figs 2B and 3A). In arguably slightly more advanced specimens, the 5HT-lir signal of the more medially located apical neuron becomes weaker and eventually ceases to display 5HT-lir during further development.

Both of the caudal neurons and the medio-ventral apical 5HT-lir neuron display 5HT-lir at *2-dpf* (Fig 3B). The dorsal branches of the longitudinal neurites around the midgut have made connection on the dorsal side of the larva forming a dorsal neurite arc (Fig 3B). A dorsally located, longitudinal 5HT-lir neurite signal extends from the more dorsally located, caudal 5HT-lir neuron anteriorly. Along its course it reaches to the dorsal neurite arc and possibly even further (Fig 3B). The signal is very short-lived: In presumably more advanced stages the signal has disappeared posteriorly (Fig 2C). In these advanced stage specimens, the early paired longitudinal neurites have ceased to display 5HT-lir in their posterior extension as well (Fig 2C). A ventral connection of neurites is detectable forming a continuous ring with the dorsal neurite arc. Its position, anterior of the mouth opening, indicates that this neurite ring might be the first 5HT-lir signal of the ring-shaped brain (Figs 2C and 3B). A pair of comparably large neurons, the first 5HT-lir neurons of the brain-ring, is subsequently showing near the ventral part of the neurite ring (Figs 2C and 3B). On the level of the mouth opening and posterior to it, there are some irregularly distributed 5HT-lir neurons detectable peripherally in the epidermis (Fig 2C). While a few of them are paired, they are not detected consistently in every specimen. Whether these signals are neurons associated with the peripheral plexus or whether they contribute to the definite nervous system could not be determined. The remaining first-formed neurons exhibit a somewhat patchy, irregular 5HT-lir signal that is arguably indicative of their degeneration. The signals of the process to the epidermal surface have become weaker (Fig 2C).

At the age of *3-dpf*, all of the remaining first-formed neurons have ceased to display 5HT-lir (Figs 2D and 3C). The peripheral 5HT-lir neurons have also become inconspicuous. In their place, just proximal of the epidermal cells, a plexus of irregularly arranged, 5HT-lir neurite signals is detected (Fig 2D). The 5HT-lir neurites of the brain-ring are becoming more prominent and numerous, especially in its ventral portion (Fig 2D). Up to two pairs of ventral and two pairs of dorsal neurons associated with the brain-ring display 5HT-lir (Figs 2D and 3C). The ventral pairs of signals are spherical to ovate. The more medially situated pair is relatively smaller than the more laterally located pair. The dorsally located neurons are pyriform. Their narrow ends point towards the dorsal brain-ring neurites (Fig 3C). In an apical position, frontal to the brain-ring, several irregular, elongated 5HT-lir signals are present (Fig 2D). They may be 5HT-lir receptor cells or precursors of 5HT-lir neurons migrating from the frontal epidermis to the brain-ring. Although not being the most prominent 5HT-lir signals, these apically located signals subsequently increase in number during further development (Fig 1C and 1D)., In older stages, on the other hand, the apical 5HT-lir signals are more reminiscent of apically located, 5HT-lir gland cells not associated with brain development. This is indicated by spherical 5HT-lir signals observed in older stages outside of the larva, which look similar to discharged glandular content (Fig 1C and 1D).

**Fig 4:**
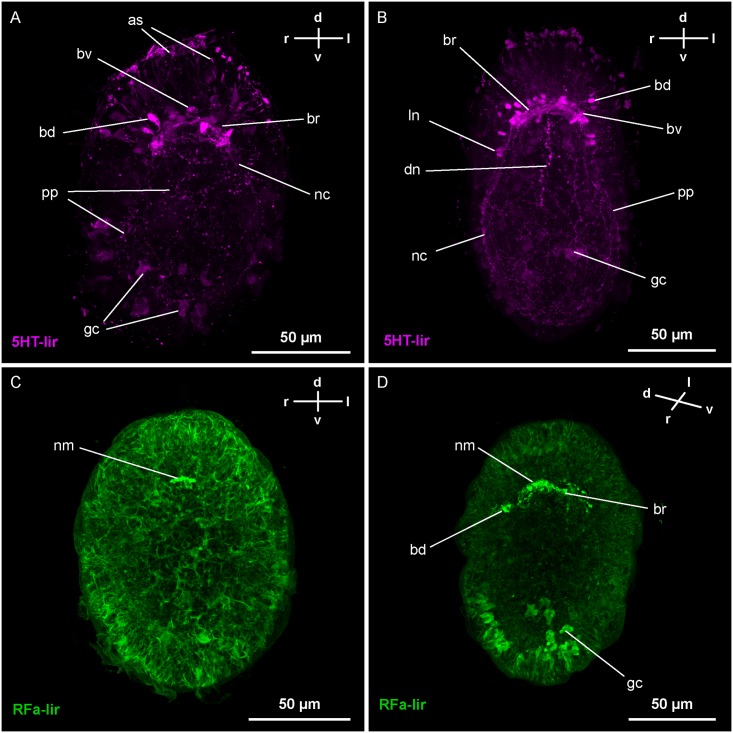
Z-Projections of immuno-stained specimens of *Carinina ochracea*. Orientation indicated by compass (d, dorsal; l, left; r, right; v, ventral); apical is up. A and B: serotonin-like immunoreactivity (*5HT-lir*). A: Larva at four days post-fertilization (*4-dpf*) (γ: 0.9). The number of neurites and neurons of the brain, displaying 5HT-lir, have increased both ventrally (*bv*) and dorsally (*bd*). From the ventral portion of the brain-ring neurite bundle (*br*) the first, weak 5HT-lir neurites of the lateral nerve cords (*nc*) are detectable. 5HT-lir signals of the peripheral plexus (*pp*) are visible as well as numerous 5HT-lir apical signals (*as*). Note the weak unspecific signals of epidermal mucus gland cells (*gc*) B: Larva at *7-dpf* (γ: 0.69). The neurites of the brain-ring (*br*) and the associated brain neurons (*bv* and *bd*) have further increased in number. The 5HT-lir dorsal neurite signals (*dn*) and neurite signals of the lateral nerve cords (*nc*) are extended posteriorly. More anteriorly located neurons (*ln*), associated with the lateral nerve cords, display 5HT-lir. 5HT-lir is also visible in the peripheral plexus (*pp*), the apical signals (*as*), and, albeit weak and unspecific, in some epidermal mucus gland cells (*gc*). C and D: FMRFamide-like immunoreactivity (RFa-lir). C: Larva at *2-dpf* (γ: 0.96). The first RFa-lir signal detectable is a neurite-like mass (*nm*) located dorso-medially, anterior of the mouth opening. D: Larva at *3-dpf* (γ: 1). The first pair of large, dorsal, RFa-lir neuron signals (*bd*) is visible. Their neurite signals project into the dorsal neurite-like mass that is continuous with the first RFa-lir neurites of the developing brain-ring (*br*). Note the unspecific signals of epidermal mucus gland cells (*gc*).

At *4-dpf*, the brain-ring is further elaborated, especially in its ventral part. It now comprises several neurite signals displaying 5HT-lir. The number of neuronal signals associated with the brain-ring on its ventral side has increased to three pairs (Fig 4A). Compared to *3-dpf* stages, all neurons seemingly have diminished in size (Figs 2D and 4A). The number of 5HT-lir neurons associated with the dorsal portion of the brain-ring is initially two but increases to three in more advanced stages (Fig 4A). Unlike the ventral neurons, which are distributed more evenly along the extension of the brain-ring neurite bundle, the dorsal neurons form two paired groups lateral of the midline. Posterior of the brain-ring, the first 5HT-lir signals of the lateral nerve cord neurites become detectable in ventro-lateral positions on each side. At this stage, they are still very inconspicuous and can be followed only for a short distance posterior of the brain-ring (Fig 4A). Along with the neurites of the lateral nerve cords, the first pair of neurons of the lateral nerve cords is faintly visible in more advanced stages. The neurons of a pyriform shape are located in close proximity to the brain-ring. The peripheral plexus of the larva displays distinct 5HT-lir signals that are seemingly more numerous than in *3-dpf* larvae (Figs 2D and 4A).

**Fig 5:**
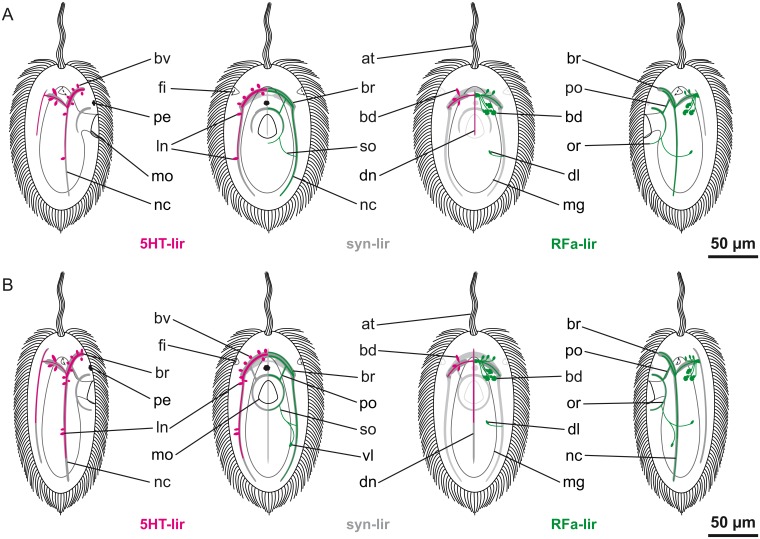
Schematic representation of nervous system development of *Carinina ochracea*. Serotonin-like (*5HT-lir*) and FMRFamide-like (*RFa-lir*) immunoreactivity are color coded in magenta and green respectively; the general nervous system architecture as outlined by synapsin-like immunoreactivity (*syn-lir*) is shown in grey. All larval stages are drawn to scale with apical tuft (*at*), epidermal cilia, fronto-lateral epidermal invaginations (*fi*), midgut (*mg*), mouth opening (*mo*), and the pigmented larval eye (*pe*) serving as landmarks (cilia of the frontal epidermal invaginations, the stomodaeum, the midgut, and epidermal cilia in the aspect of view omitted), apical is up. For clarity, the peripheral plexus is not shown. A and B: from left to right: lateral view from right, ventral view, dorsal view, lateral view from left. Since the nervous system is roughly symmetrical only one side is shown (left: 5HT-lir, right: RFa-lir). A: Larva at five days post-fertilization (*5-dpf*). B: Larva at *7-dpf*. Further explanations, see text.–*bd*, dorsal brain neuron; *br*, brain-ring; *bv*, vental brain neuron; *ln*, lateral nerve cord neuron; *dl*, dorso-lateral neuron; *dn*, dorsal nerve; *nc*, lateral nerve cord; *or*, oral ring neurite bundle; *po*, pre-oral neurite bundle; *so*, sub-oral neurite; *vl*, ventrolateral neuron.

More 5HT-lir neurites are detectable in the brain-ring, along with neuritic concentrations in the area where the lateral nerve cords branch of from the brain-ring after *5-dpf* (Fig 1C). There are two additional pairs of 5HT-lir neurons associated with ventral arc of the brain-ring, thus amounting to a total of five pairs (Figs 1C and 5A). The number of 5HT-lir neurons associated with the dorsal brain-ring remains unchanged. A single, longitudinal 5HT-lir neurite signal is detected in a dorso-median postion. It extends from the brain-ring neurite bundle posteriorly to about the level of the mouth opening (Figs 1C and 5A). This signal arguably represents the first 5HT-lir neurite signal of the dorsal nerve. The neurite bundles of the lateral nerve cords have become slightly more prominent. An additional pair of 5HT-lir neurons, associated with the lateral nerve cord neurites, is visible, situated just posterior of the mouth opening (Figs 1C and 5A).

During further development, the 5HT-lir neurites in the brain-ring become more numerous (Figs 4B and 5B). At *7-dpf* a 6^th^ pair of neurons is detectable along the ventral portion of the brain-ring while the number of dorsal neurons remains unchanged (Figs 4B and 5B). Both the neurites of the dorsal nerve and the lateral nerve cords are elongated posteriorly, but the latter are still comparably inconspicuous (Figs 4B and 5B). Additional pairs of neurons are detectable associated with the anterior part of the lateral nerve cords (Figs 4B and 5B). A second pair of neurons associated with the lateral nerve cords more posteriorly is feebly displaying 5HT-lir. It is located in the vicinity of the first formed pair posterior of the mouth opening (Fig 5B).

Up to *10-dpf*, the 5HT-lir neurite of the dorsal nerve extends further frontally past the neurites of the dorsal portion of the brain-ring (Fig 1D). The number of 5HT-lir neurons associated with the ventral arc of the brain-ring increases to seven or eight pairs. In a few larvae, an additional pair of 5HT-lir dorsal neurons can be seen. In the most advanced-stage larvae, 5HT-lir is displayed in the brain-ring, the lateral nerve cords, the dorsal nerve, and the peripheral plexus. The 5HT-lir signals of numerous apical epidermal cells are still faintly visible (Fig 1D).

### Development of the FMRFamide-like immunoreactive neural elements

The first RFa-lir signal appears at 2-*dpf*. The most prominent signal is a dense mass of RFa-lir signals located on the dorso-median side of the larva at the level anterior of the mouth opening. The signals, oriented transversely with respect to the longitudinal axis of the larva, are likely a tangle of RFA-lir neurites (Figs 3B and 4C). Associated RFa-lir perikarya are not evident.

At *3-dpf*, a pair of neurites is present that extends from the dense neurite-like mass on either side around the circumference of the larva at the level of the future brain-ring (Figs 3C and 4D). Along the ventral brain-ring neurites conspicuous varicosities are visible. They can be distinguished from neuronal perikarya by the absence of an unstained, i.e. dark spherical structure representing the nucleus in neuronal perikarya. The dense mass of RFa-lir signals is enlarged. Supposedly, it is composed of more neurites. A pair of comparably large neurons is situated dorso-laterally on both sides just posterior to the fist brain-ring neurites (Figs 3C and 4D). The neurons have an irregularly lobate outline, and each is connected to the dense dorso-median neurite mass by a single neurite.

**Fig 6:**
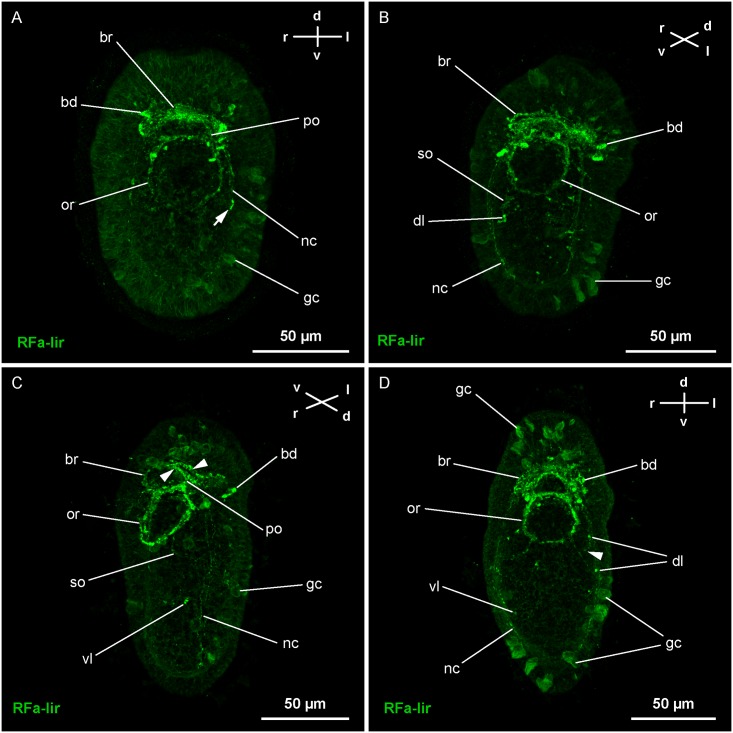
Z-Projections of immuno-stained specimens of *Carinina ochracea*. Orientation indicated by compass (d, dorsal; l, left; r, right; v, ventral); apical is up. FMRFamide-like immunoreactivity (RFa-lir). A: Larva at four days post-fertilization (*4-dpf*) (γ: 0.93). The neurites of the brain have become more numerous and form a closed ring (*br*). There are comparably large dorsal RFa-lir neurons (*bd*) associated with the brain-ring. From the ventral part of the brain-ring the first RFa-lir neurites of the lateral nerve cords (*nc*) extend posteriorly. Around the mouth opening RFa-lir neurite signals (*or*) start to form along with weak RFa-lir pre-oral neurite signals (*po*) connecting the oral ring neurite with the ventral part of the brain-ring. Note the putative growth cone signals associated with the left lateral nerve cord (arrow) B: Larva at *5-dpf* (γ: 0.87). The RFa-lir neurites of the brain-ring (*br*) and the associated dorsal neurons (*bd*) have increased in number. The same is true for the oral ring neurites (*or*) and the neurites of the lateral nerve cords (*nc*) that are also considerably extended posteriorly. A pair of RFa-lir sub-oral neurites (*so*) connects the posterior of the oral ring with paired dorsolateral neurons (*dl*). C: Larva at *7-dpf* (γ: 1). The RFa-lir neurites of the ventral part of the brain (*br*) are arranged in two concentrated bundles (arrowheads) and several, putatively individual neurites. The RFa-lir neurites of the oral ring (*or*) have become considerably stronger and the neurites of the lateral nerve cords (*nc*) extend further posteriorly. In their vicinity and proximal of them paired ventro-lateral neurons (*vl*) display RFa-lir. The RFa-lir pre-oral neurite bundle (*po*) that is connecting the ventral part of the brain-ring with the oral ring has become more distinct. The RFa-lir neurons associated with the dorsal part of the brain (*bd*) show a conspicuous, lobate shape. D: Larva at *10-dpf* (γ: 1). The RFa-lir neurites in the brain-ring (*br*), and the oral ring neurite bundle (*or*) have increased in number and have become more distinct. The neurites of the lateral nerve cords (*nc*) seem to remain unchanged. Additional neurons displaying RFa-lir are found associated with the dorsal part of the brain-ring (*bd*). An additional pair of dorso-lateral neurons (*dl*) anterior of the first pair is detectable associated with a branch of the suboral neurite bundle (arrowhead).–*gc*, mucus gland cells.

The number of neurons displaying RFa-lir on the dorsal side of the brain-ring increases during subsequent development. At least one additional pair of comparably large, lobate neurons is observable posterior to the brain-ring at *4-dpf* (Fig 6A). Each neuron projects into the dense dorsal neurite-like mass via an individual neurite. Dorsally, anterior of the brain-ring, one pair of smaller, pyriform, dorso-laterally located RFa-lir neurons is visible. Like their posterior counterparts, each of the anterior neurons is connected by a neurite to the dorso-median neurite mass. The lateral nerve cords exhibit few RFa-lir neurites that extend posteriorly to the level of the posterior rim of the mouth opening (Fig 6A). In some specimens, club-shaped signals, displaying RFa-lir, are seen at the posterior-most end of the lateral nerve cords (Fig 6A). Their comparably small size makes it likely that these signals are growth cones of lateral nerve cord neurites. The mouth opening is encircled by an RFa-lir neurite signal that is not completely closed in its posterior-most part (oral ring neurite bundle). In its anterior semicircle the signal is stronger with putative varicosity-like signals antero-laterally associated with it (Fig 6A). The paired pre-oral neurite bundles, connecting the oral ring neurite bundle to the RFa-lir signal of the ventral brain-ring, weakly display RFa-lir (Fig 6A).

At *5-dpf*, the signal strength of the brain-ring is increased in its ventral portion, forming two more prominent neurite bundles along with several, probably individual, neurites (Figs 5A and 6B). In the dorsal part of the larva the number of posterior and anterior neurons is increased to approximately three pairs and two pairs respectively (Fig 5A). Along the ventral part of the brain-ring additional small RFa-lir signals are detectable, likely varicosities (Fig 6B). The oral ring neurite bundle is completely closed, although its signal is still stronger in the anterior portion (Figs 5A and 6B). The signals of the lateral nerve cord neurites have increased in number and are extended to almost the full length of the larvae. However, the neurites are not observed to be connected posteriorly by forming an anal commissure (Figs 5A and 6B). A pair of pyriform neurons is situated dorso-laterally on both sides, at half of the length of the larva (Fig 6B). Each neuron sends out a neurite that extends toward the ventral side of the body. The neurite traverses the nerve cord neurite bundle proximally on its respective side at around half of the length of the nerve cord. More ventrally, the neurites are bending to the anterior to connect to the posterior part of the oral ring neurite bundle (Fig 5A). These neurites are herein termed suboral neurites.

At *7-dpf*, the signal of the oral ring is evenly strong throughout, most likely comprising a bundle of neurites (Figs 5B and 6C). The same applies to the brain-ring neurites and the pre-oral neurite bundle, the former still showing two more distinct bundles in its ventral part (Figs 5B and 6C). The number of putative posterior neurons situated anterior of the brain-ring has increased to three pairs, while the number of posteriorly located neurons apparently stays unchanged (Fig 5B). The signals of the lateral nerve cords seem to remain unchanged, although they elongate as the larva elongates (Figs 5B and 6C). In the posterior third of the larva a pair of pyriform, comparably small, neurons displays RFa-lir (Figs 5B and 6C). The neurons are located ventro-laterally on each side in close vicinity to the lateral nerve cords, but proximal of them. An RFa-lir neurite extends from each neuron anteriorly to fuse with the suboral neurite of the respective body-side at about half of its length (Fig 5B).

The number of RFa-lir neurites in the brain-ring has further increased at *10-dpf* (Fig 6D). One posteriorly, and two anteriorly located additional pairs of neurons are detected associated to its dorsal part. In the ventral part of the brain-ring the number of varicosities seems increased as well. The number of neurites in the oral ring neurite bundle, the pre-oral neurite bundle, and the lateral nerve cords has increased slightly (Fig 6D). An additional pair of dorso-lateral neurons displaying RFa-lir, are located anterior of the sub-oral neurites and their associated perikarya (Fig 6D). Their neurites span the dorso-lateral sides of the larva and associate with the suboral neurite bundles ventral of the lateral nerve cords.

In the most advanced-stage larvae neurites displaying RFa-lir are found in the brain, the lateral nerve cords, and the esophageal nervous system. The esophageal nervous system comprises the pre-oral neurite bundles, the oral ring neurite bundle, and the sub-oral neurites. Neurons are only unambiguously detectable associated with the ventral-most portion of the brain-ring and the lateral nerve cords.

## Discussion

### Immunoreactivity of the nervous system of *Carinina ochracea*

The nervous system of adult animals of *Carinina ochracea* has been investigated by means of IF/CLSM [27,31]. Immunoreactivity against acetylated α-tubulin, serotonin, and FMRFamide antibodies has been shown to be displayed as specific signals in the central and the peripheral nervous system [27,31]. Antibodies against synapsin (SYNORF1 = 3C11) [25] have so far not been widely used in studies on the development of the nervous system in Spiralia [32–35]. Furthermore, syn-lir has been reported to be problematic in certain spiralian taxa: signals specific to neuropil have been detected in Cycliophora and Myzostomida [34,35], while no specific staining could be found in some investigated annelid taxa (Polychaeta and Echiura) [32,33]. Immunoreactivity against synapsin antibodies has not been comprehensively assessed before in Nemertea. In *C*. *ochracea*, syn-lir signals are exclusively detected in structures that also display neurite-like tub-lir. Therefore, it is reasonable to assume that syn-lir is specific to the synaptic portions of the nervous system (i.e. neuropil, following the definition of [36]). In the majority of structures of the nervous system, syn-lir is usually observed later than tub-lir, indicating progress in circuitry development in the respective part of the nervous system.

### Architecture of the developing nervous system in *Carinina ochracea*

Early elements of the nervous system, like the apical and caudal neurons and longitudinal neurites, have repeatedly been reported to display tub-lir and/or 5HT-lir in various spiralian species [6]. While 5HT-lir is also displayed in the respective neural elements of young *C*. *ochracea* larvae, tub-lir is not consistently observed. More often than not, no tub-lir signals could be detected in the first-formed apical and caudal neurons. This may either be due to the strong tub-lir signals of cilia of the adjacent epidermis, or to the presumably short-lived nature of tubulin expression in the respective early neurons. Neither apical nor caudal transitory RFa-lir signals were detected in *C*. *ochracea*. All nervous system specific RFa-lir elements detected are retained and elaborated during further development. In advanced developmental stages of *C*. *ochracea*, tub-lir displays neurite-like signals in all parts of the nervous system. The most conspicuous nervous system components are the ring-shaped brain and the paired lateral nerve cords. The tub-lir signals produced by the microtubules of the ciliary axonemes that are in close proximity to the nervous system partly obscure the finer structures of the nervous system shown by tub-lir. Finer structures include the dorsal nerve, the esophageal component of the nervous system, and the peripheral plexus. Like in adult specimens, the tub-lir signals of the brain-ring, the paired, lateral nerve cords, the dorsal nerve, and the esophageal nervous system (i.e. oral ring neurite bundle and pre-oral neurite bundles) all seem to be situated within the epidermis (i.e. in an basiepidermal position) [27]. The proboscis apparatus has not yet developed [37], hence its nervous system is also absent. The majority of the nervous system components display 5HT-lir and RFa-lir. Interestingly, the brain in developmental stages seemingly displays neuronal RFa-lir signals merely in the dorsal part, while 5HT-lir perikarya are found in both the ventral and in the dorsal portion of the brain-ring. Furthermore, while 5HT-lir is absent from the esophageal nervous system, RFa-lir is absent from the dorsal nerve and the peripheral plexus. The peripheral plexus in the larva corresponds in position to the epidermal plexus of the adult. However, the arrangement of the neurites of the epidermal plexus in the adult (ladder-like) [27] is different from the arrangement in the larva (diffuse, net-like). Another difference between the larval and the adult epidermal plexus is the expression of RFa-lir in the latter that is not observed in the former. The observed differences indicate that, although in the same position relative to the epidermis, the larval and the adult plexus might not be identical. Alternatively, the larval peripheral plexus might be substantially transformed to the adult configuration during the transition from pelagic to benthic life.

The above stated findings indicate that there are only few transitory elements observable in *C*. *ochracea*, namely the apical and caudal 5HT-lir neurons along with their 5HT-lir neurites. The remainder of the nervous system observed in the larva is likely identical with the nervous system of the adult. Therefore, the persisting components of the larval nervous system have to be considered as the developing definite nervous system of the adult. In *C*. *ochracea*, the definite CNS develops in a strictly anterior to posterior progression. The majority of the observed neurons of the brain apparently migrate inward from the frontal epidermis, not exactly at, but in the vicinity of the apical pit.

### Comparative aspects of nervous system development in Nemertea

Although the largest body of IF/CLSM data on the developing nervous system is available for the pilidium larva [14,16,17], it is now evident that this larva is not typical of Nemertea [22]. In the pilidium larva, a transitory larval epidermis with associated neurons and muscles is formed, most likely due to prolonged, planktotrophy [19,21,22]. During the catastrophic metamorphosis, the larval envelope is shed and eaten by the juvenile [16,38,39]. Therefore, the larval nervous system should be regarded as highly derived. The derived nature of the hoplonemertean larva has recently been acknowledged, and it has consequently been renamed “decidula larva” [22,23,40,41]. However, unlike in the pilidium larva, the transitory larval envelope in the decidula larva is restricted to several epidermal cells that are shed while the nervous system is retained and elaborated [12,13,15,18,40,41]. Thus, development of the hoplonemertean larva seems more similar to the mode observed in the basally branching palaeonemertean species [10,13,22,42].

Two apical 5HT-lir neurons, reminiscent of the first-formed apical neurons found in *C*. *ochracea*, have been described in the hoplonemertean species *Quasitetrastemma stimpsoni* and *Pantinonemertes californiensis* [15,18]. However, in the hoplonemertean species additional, ventro-laterally located, flask-shaped, so-called “subapical” neurons with contact to the fronto-lateral epidermis are detected. Proximal of the flask-shaped apical neurons one (*Q*. *stimpsoni*) or two (*P*. *californiensis*) roughly spherical, so-called “additional” apical neurons are described, which have no equivalent in *C*. *ochracea* [15,18]. From the data at hand, it is impossible to evaluate whether the subapical and additional apical neurons are ancestral in Nemertea and consequently reduced in *C*. *ochracea*, or apomorphic in Hoplonemertea. The early apical 5HT-lir signals cease to be detectable at the onset of brain development in *C*. *ochracea*, while they persist through the onset of brain development in the hoplonemertean species [15,18]. In *P*. *californiensis* all apical and subapical neurons disappear at the onset of development of the lateral nerve cords [18], while in *Q*. *stimpsoni* most neurons, except for the single additional apical neuron, seem to be incorporated into the developing brain [15]. In contrast to *C*. *ochracea*, only one caudal neuron is detected in decidula larvae [15,18]. The existing data do not allow for a sound hypothesis to be stated on the evolution of the second caudal neuron. This neuron is either apomorphic in *C*. *ochracea*, or it represents an ancestral trait that has been reduced in the last common ancestor of Hoplonemertea. A pair of anteriorly projecting neurites is reported from the caudal neuron in *P*. *californiensis* [18]. The same is seen in the more ventrally located caudal neuron of *C*. *ochracea*, indicating their homology. The caudal neuron and its caudal neurites are lost before the neurites of the lateral nerve cords are detectable in *C*. *ochracea* and *P*. *californiensis* [18]. In *Q*. *stimpsoni*, on the other hand, the caudal neuron disappears later, after the formation of the first lateral nerve cord neurites [15]. The presence or absence of apical or caudal transitory RFa-lir neurons in decidula larvae remains to be investigated.

The first element of the definite nervous system detectable in *2-dpf* stages of *C*. *ochracea* is a 5HT-lir peripheral nerve plexus. There is no specific description regarding the peripheral plexus in hoplonemertean species. However, images of early and late rudiment stages of the larva of the hoplonemertean *P*. *californiensis* [18] show signals similar to those reported here for the plexus of *C*. *ochracea*. A 5HT-lir nerve plexus has also been described in pilidium larvae [16,17]. However, since it is shed along with the larval envelope during metamorphosis, this plexus should be regarded as a purely larval structure. Data on the development of the prospective adult epidermal plexus are lacking.

In all nemertean species investigated by means of IF/CLSM, development of the CNS is quite uniform. The first immunoreactive elements attributable to the CNS are found in the future brain-ring. From there, differentiation of the nerve cords proceeds towards the posterior. In none of the species examined a segmental pattern of the nervous system formation was observed [14–18]. There is, however, considerable disagreement regarding the origin of the neuronal precursor cells of the brain. Commonly, a pair of fronto-lateral, epidermal invaginations is interpreted as precursor of the brain and lateral nerve cords in palaeonemertean and most hoplonemertean species [12,13,24,42]. More recently however, the same epidermal invaginations have been interpreted as the primordia of the cerebral organs in a hoplonemertean species [40]. Corresponding epidermal invaginations have also been described in the larva of *C*. *ochracea* [37]. According to the data presented herein, there is no neuron-specific immunoreactivity against any of the employed antibodies detectable in the fronto-lateral, epidermal invaginations. In contrast, in the hoplonemertean species investigated by means of IF/CLSM, there are neurons connected to the fronto-lateral epidermal invaginations, the 5HT-lir subapical neurons. As outlined above, however, these subapical neurons are not part of the developing definite nervous system [15,18]. In various mollusk species, similar invaginations have been shown to give rise to the cerebral ganglia [43–45], also without displaying immuoreactivity against the respective antibodies [46–48]. While the hypothesis that the fronto-lateral epidermal invaginations represent the primordia of the brain-ring seems to be supported by published information, it is challenged by the fact that at least RFa-lir neurons of the brain likely originate from more dorsally located parts of the frontal epidermis.

The available comparative information results in the following ancestral pattern of the early 5HT-lir nervous system of nemertean larvae: A minimum of two apical flask-shaped 5HT-lir neurons are present in the vicinity of the apical pit, along with a single caudal flask-shaped 5HT-lir neuron with paired lateral neurites projecting to the anterior. All listed structures are lost before or shortly after the onset of the development of the brain. A 5HT-lir trochal neurite bundle is absent. Before the prospective adult CNS becomes evident, a peripheral 5HT-lir peripheral plexus develops, which is arguably retained as the adult epidermal plexus. The brain is formed from cells migrating inward from the frontal epidermis. The contribution of the paired fronto-lateral epidermal invaginations to brain development is not yet entirely clear (see discussion above and in [18,40,41]). Paired unsegmented, laterally located, nerve cords grow out posteriorly from the ventral portion of the brain-ring, completing the adult CNS. Nevertheless, the limited number of detailed, comprehensive accounts on nervous system development in Nemertea, together with the unclear systematic status of the palaeonemertean species [19,20,49,50], renders the ancestral state reconstruction preliminary. Clearly, more data on the palaeonemertean lineages and on immunoreactivity against other nervous system specific antibodies is needed to fruitfully address the unsettled questions outlined above.

## Conclusions

While the ring-shape of the brain is an apomorphy of Nemertea the remainder of the CNS forms as one pair of unsegmented, lateral nerve cords. Thus, the architecture of the definite CNS in Nemertea is congruent with the hypothetical ancestral spiralian pattern that consists of an anterior brain and paired, unsegmented, possibly lateral nerve cords [7,51]. The occurrence of a transitory caudal neuron in Nemertea supports the recently proposed hypothesis that posterior, possibly transitory neurons is an ancestral trait in the development of nervous systems in Spiralia (see discussion in [48]). A trochophore type larva has been postulated in the last common ancestor of Spiralia (in the sense of [1,52]) [2,4,53,54]. It has been hypothesized to have possessed a simple apical organ that was restricted to the larval phase [2,4,7]. In the last common ancestor of Nemertea, an apical organ was likely present in the form of at least two apical, flask-shaped, transitory 5HT-lir neurons in the vicinity of the apical pit. Furthermore, a trochal neurite bundle, underlying the prototroch, has been hypothesized in the larva of the last common ancestor of Spiralia [4,7,36,55]. In the light of this hypothesis, the absence of the trochal neurite bundle in nemertean species has to be interpreted as due to secondary reduction in their last common ancestor. This reduction may be explained by the predatory feeding in Nemertea, which is still found in some larvae of extant nemertean species [23,56]. An alternative hypothesis, supported by most recent phylogenetic analyses, regards Lophotrochozoa (in the sense of [1]) as an in-group of directly developing spiralian taxa [51,57,58]. As a consequence, this hypothesis calls into question the ancestral nature of the pelagic, feeding larva in Spiralia (in the sense of [1,52]). Accordingly, the prototroch with its associated trochal neurites represents a derived trait of lophotrochozoan species. Provided a basal position of Nemertea as sister-group to the remaining lophotrochozoan clades [59,60], the absence of the trochal neurite bundle would be more parsimoniously explained as representing the primary condition.

## Supporting Information

S1 Appendix DNA Extraction, PCR, nucleotide sequencing, and BLAST of COI “barcode-region” for confirmation of the identification. (DOCX)

S2 Appendix Image processing protocol. (DOCX)

S1 Fig Z-Projections of immuno-stained specimens prior to image adjustments (see S2 Appendix). (TIF)

S1 Table List of variable settings in image acquisition and image processing (see S2 Appendix). (DOCX)
