# Correction: Development of the Nervous System of *Carinina ochracea* (Palaeonemer-tea, Nemertea)

**DOI:** 10.1371/journal.pone.0169477

**Published:** 2016-12-29

**Authors:** The PLOS ONE Staff

The word “Palaeonemertea” is misspelled in the article title. The correct title is: Development of the Nervous System of *Carinina ochracea* (Palaeonemertea, Nemertea). The correct citation is: von Döhren J (2016) Development of the Nervous System of *Carinina ochracea* (Palaeonemertea, Nemertea). PLoS ONE 11(10): e0165649. doi:10.1371/journal.pone.0165649. The publisher apologizes for the error.
