# Supplementary material for: Development of the Nervous System of Carinina ochracea (Palaeonemer-tea, Nemertea)
Source: PLoS One. 2016 Oct 28;11(10):e0165649. doi: 10.1371/journal.pone.0165649 (PMC5085047; doi:10.1371/journal.pone.0165649)
Supplement: S1 Appendix — (DOCX) [file pone.0165649.s001.docx]

**S1 Appendix. DNA Extraction, PCR, nucleotide sequencing, and BLAST of COI “barcode-region” for confirmation of the identification**

Tissue from adult animals of the same population as those used for obtaining gametes was fixed in 99.8% ethanol. Extraction of DNA was performed using the DNeasy Blood and Tissue kit (Qiagen) following the manufacturer’s protocol. For amplification of the so-called “barcode-region” of the mitochongrial COI gene the standard primer pair LCO1490/HCO2198 (Metabion) was used [1]. Taq-Polymerase (0.25µl 5U/µl, Invitrogen) was used in a total volume of 50µl (40.25 µl distilled water, 5µl 10x Mg^2+^buffer, 1µl 10mM dNTPs, 1.5µl 50mM MgCl_2_, 1µm 1:10 primers, and 1µl sample DNA). Thermal cycling was initated by 2 min of denaturation at 94° C, followed by 40 cycles of 30 sec of denaturation at 94° C, 1 min of annealing at 45° C, 1min of elongation at 72° C, and 1 min of final extension at 72° C. PCR-reaction products were visualized on a 1.5% agarose gel along with 3µl of 2-log DNA-ladder (New England BioLabs). Purification of PCR-reaction product was done with the aid of the NucleoSpin Extract II kit (Macherey & Nagel) following the manufacturer’s instructions. The sequencing of purified PCR-reaction product (10µl and 4µl 1:10 primer-mix, only one primer was added) was performed after the Sanger method (Agowa). Identification of the species was done by BLAST search of sequences from 17 individuals with the default settings, as implemented on the NCBI website (<http://www.ncbi.nlm.nih.gov/>).

**References**

1. Folmer O, Black M, Hoeh W, Lutz R, Vrijenhoek R. DNA primers for amplification of mitochondrial cytochrome c oxidase subunit I from diverse metazoan invertebrates. Mol Mar Biol Biotechnol. 1994;3: 294–299. doi:10.1371/journal.pone.0013102
