# Supplementary material for: Development of the Nervous System of Carinina ochracea (Palaeonemer-tea, Nemertea)
Source: PLoS One. 2016 Oct 28;11(10):e0165649. doi: 10.1371/journal.pone.0165649 (PMC5085047; doi:10.1371/journal.pone.0165649)
Supplement: S2 Appendix — (DOCX) [file pone.0165649.s002.docx]

**S2 Appendix. Image Processing Protocol**

**Pre-processing**

1. Image🡪Duplicate… (for range of duplicated slices, see Table S1)

2. Image🡪Transform🡪Rotate… (Interpolation: none; for angle of rotation, see Table S1)

3. Image🡪Transform🡪Translate… (Interpolation: none; for degree of translation, see Table S1)

4. Image🡪Color🡪Split Channels

5. *for tub-lir:* Image🡪Lookup Tables🡪Cyan; *for syn-lir:* Image🡪Lookup Tables🡪Grays; *for 5HT-lir:* Image🡪Lookup Tables🡪Magenta; *for RFa-lir:* Image🡪Lookup Tables🡪Green

**Image Adjustments**

*for 5HT-lir (≥2-dpf); syn-lir (≥3-dpf):*

1. Process🡪Image Calculator: channel1(5HT-lir)-stack – Min – channel2(syn-lir)-Stack 🡺 Res-stack1

2. Process🡪Image Calculator: channel2-stack – Substract – Res-stack1 🡺 Res-stack2

3. Process🡪Image Calculator: channel1-stack – Substract – Res-stack2 🡺 Res-stack3 🡺 **5HT-lir**

4. Process🡪Image Calculator: channel1-stack – Difference – channel2-Stack 🡺 Res-stack4

5. Process🡪Image Calculator: Res-stack1 – Difference – Res-stack4 🡺 Res-stack5

6. Process🡪Image Calculator: channel2-stack – Difference – Res-stack5 🡺 Res-stack6

7. Process🡪Image Calculator: channel2-stack – Substract – Res-stack6 🡺 Res-stack7 🡺 **syn-lir**

*for RFa-lir:*

8. Process🡪Substract Background (Rolling ball radius: 30 pixels; other settings default)

**Post-Processing**

1. *for tub-lir:* Process🡪Math…🡪Max (Value: 93)

2. *for all:* Process🡪Math🡪Gamma… (Value; see Table S1)

3. Image🡪Stacks🡪Z Project… (All slices; Projection type: Maximum Intensity)

4. Image🡪Adjust🡪Brightness/Contrast… set background to 0 (blue in HiLo Indicator LUT; details see Table S1)

5. Process🡪Enhance Contrast (Saturated pixels: 0.2%, Normalize checked; details see Table S1)

6. *for 5HT-lir & syn-lir channels at 5-dpf and 10-dpf:*

Image🡪Color🡪Merge Channels… (5HT-lir: magenta, syn-lir: grays)

7. *for all:*

Image🡪Type🡪RGB Color
