# Supplementary figures and images for: Development of the Nervous System of Carinina ochracea (Palaeonemer-tea, Nemertea)
Source: PLoS One. 2016 Oct 28;11(10):e0165649. doi: 10.1371/journal.pone.0165649 (PMC5085047; doi:10.1371/journal.pone.0165649)

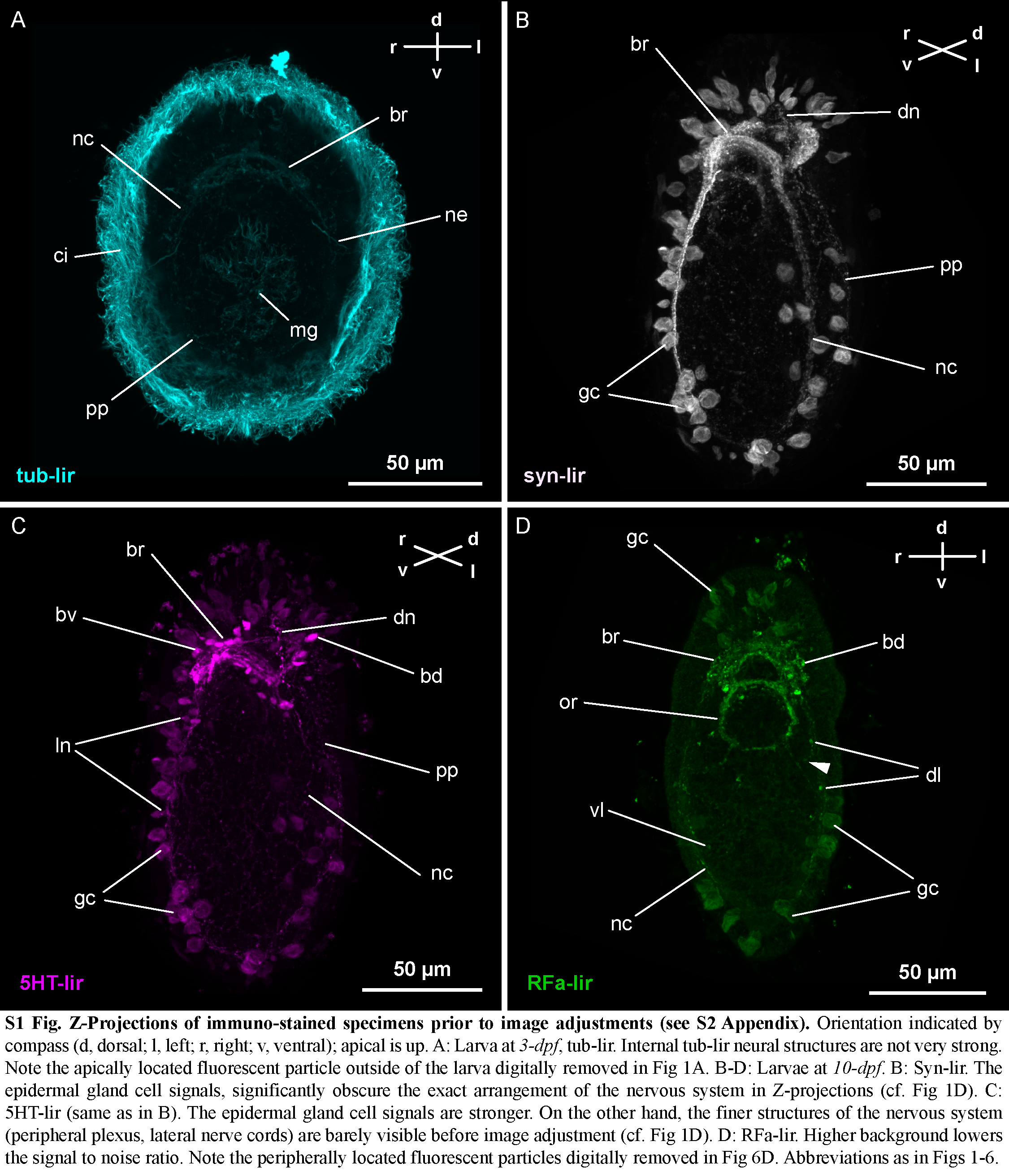

Supplement: S1 Fig — (TIF) [file pone.0165649.s003.tif]
