# Supplementary material for: Development of the Nervous System of Carinina ochracea (Palaeonemer-tea, Nemertea)
Source: PLoS One. 2016 Oct 28;11(10):e0165649. doi: 10.1371/journal.pone.0165649 (PMC5085047; doi:10.1371/journal.pone.0165649)
Supplement: S1 Table — (DOCX) [file pone.0165649.s004.docx]

**S1 Table. List of variable settings in image acquisition and image processing (see S2 Appendix).**

| **number of image: larval age and antibody staining** | **prim/sec antibody ^1^) ( ): channel used for image processing (see Appendix S2)** | **laser-line intensity c1 (in %) ( ): λ in nm** | **laser-line intensity c2 (in %) ( ): λ in nm** | **detector gain c1 ( ): λ in nm** | **detector gain c2 ( ): λ in nm** | **detector offset**  **c1 (in %) ( ): λ in nm** | **detector offset**  **c2 (in %) ( ): λ in nm** | **emission spectrum c1 (in nm)  ( ): λ in nm** | **emission spectrum c2 (in nm)  ( ): λ in nm** | **pixel dimensions**  **(in nm)** | **duplicated (projected) slices** | **displayed channels** | **manual cleaning ^2^)** | **rotate (in °)** | **translate (in pixels)** | **gamma correction** | **contrast stretching** | **direct link to image series deposited in MorphDBase** |
| --- | --- | --- | --- | --- | --- | --- | --- | --- | --- | --- | --- | --- | --- | --- | --- | --- | --- | --- |
| **Fig 1A:** 3-*dpf*, tub-lir | tub/AF488  (5HT/AF568) | (488) 16 | --- | (488) 680 | --- | (488) 0 | --- | (488)  495-560 | --- | 168 | 18-55 (38/61) | 1 | yes | 0 | 25; 15 | 0.89 | 10-93 | www.morphdbase.de/?J_vonDoehren_20160811-M-20.1 |
| **Fig 1B:** 3-*dpf*, syn-lir | 5HT/AF488  & syn/AF568 | (488)  37 | (532)  47 | (488) 580 | (532)  670 | (488) -0.4 | (532)  -0.4 | (488)  495-560 | (532)  578-643 | 168 | 13-45 (45/59) | 2 | no | -89 | 45; -35 | 0.82 | 4-179 | [www.morphdbase.de/?J_vonDoehren_20160811-M-21.1](http://www.morphdbase.de/?J_vonDoehren_20160811-M-21.1) |
| **Fig 1C:** 5-*dpf*, 5HT- & syn-lir | 5HT/AF488  & syn/AF568 | (488) 34 | (532)  39 | (488) 600 | (532)  630 | (488) -0.4 | (532)  -0.4 | (488)  495-560 | (532)  593-637 | 168 | 5HT: 1-56 (56/60); syn: 14-55 (42-60) | 1; 3 | no | 115 | cropped | 5HT:0.89; syn:0.88 | 5HT:3-255; syn:1-235 | [www.morphdbase.de/?J_vonDoehren_20160811-M-22.1](http://www.morphdbase.de/?J_vonDoehren_20160811-M-22.1)  [www.morphdbase.de/?J_vonDoehren_20160811-M-23.1](http://www.morphdbase.de/?J_vonDoehren_20160811-M-23.1) |
| **Fig 1D:** 10-*dpf*, 5HT- & syn-lir | 5HT/AF488  & syn/AF568 | (488) 33 | (532)  32 | (488) 560 | (532)  630 | (488) -0.4 | (532)  -0.4 | (488)  495-560 | (532)  585-635 | 179 | 5HT: 2-45 (44/48); syn: 6-45 (40/48) | 1 | no | 62 | cropped | 5HT:0.74; syn:0.78 | 5HT:2-249; syn:2-237 | [www.morphdbase.de/?J_vonDoehren_20160811-M-24.1](http://www.morphdbase.de/?J_vonDoehren_20160811-M-24.1)  [www.morphdbase.de/?J_vonDoehren_20160811-M-25.1](http://www.morphdbase.de/?J_vonDoehren_20160811-M-25.1) |
| **Fig 2A:** 1-*dpf* (early), 5HT-lir | 5HT/AF488 | (488) 34 | --- | (488) 560 | --- | (488) -0.4 | --- | (488)  495-560 | --- | 149 | 7-58 (52/62) | 1 | no | -90 | -15; 15 | 0.84 | 3-201 | [www.morphdbase.de/?J_vonDoehren_20160811-M-26.1](http://www.morphdbase.de/?J_vonDoehren_20160811-M-26.1) |
| **Fig 2B:** 1-*dpf* (late), 5HT-lir | 5HT/AF568 | (532) 42 | --- | (532) 600 | --- | (532)  -0.4 | --- | (532)  595-648 | --- | 138 | 2-26 (25/36) | 1 | yes | -103 | -10; -15 | 0.77 | 6-125 | [www.morphdbase.de/?J_vonDoehren_20160811-M-27.1](http://www.morphdbase.de/?J_vonDoehren_20160811-M-27.1) |
| **Fig 2C:** 2-*dpf*, 5HT-lir | 5HT/AF488  & tub/AF633 | (488) 35 | (635) 37 | (488) 675 | (635) 650 | (488) -0.7 | (635) -0.4 | (488)  495-560 | (635)  645-690 | 179 | 8-65 (58/71) | 1 | no | -65 | 3; 5 | 0.9 | 3-164 | [www.morphdbase.de/?J_vonDoehren_20160811-M-28.1](http://www.morphdbase.de/?J_vonDoehren_20160811-M-28.1) |
| **Fig 2D:** 3-*dpf*, 5HT-lir | tub/AF488  & 5HT/AF568 | (488) 16 | (532)  37 | (488) 590 | (532)  700 | (488) 0 | (532)  0 | (488)  495-560 | (532)  585-635 | 168 | 8-59 (52/01) | 1 | yes | 25 | -40; 35 | 0.9 | 6-173 | [www.morphdbase.de/?J_vonDoehren_20160811-M-29.1](http://www.morphdbase.de/?J_vonDoehren_20160811-M-29.1) |
| **Fig 4A:** 4-*dpf*, 5HT-lir | 5HT/AF488  & syn/AF568 | (488) 31 | (532)  43 | (488) 560 | (532)  600 | (488) -0.4 | (532)  -0.4 | (488)  492-557 | (532)  565-630 | 163 | 5HT: 3-58 (56/61); syn: 14-53 (40/61) | 1 | no | -72 | 55; 15 | 0.90 | 3-255 | [www.morphdbase.de/?J_vonDoehren_20160811-M-30.1](http://www.morphdbase.de/?J_vonDoehren_20160811-M-30.1) |
| **Fig 4B:** 7-*dpf*, 5HT-lir | 5HT/AF488  & syn/AF568 | (488) 32 | (532)  32 | (488) 540 | (532)  610 | (488) -0.4 | (532)  -0.4 | (488)  495-560 | (532)  585-635 | 185 | 5HT: 7-52 (46/55); syn: 12-49 (38/55) | 1 | no | 90 | 70; -3 | 0.69 | 5-231 | [www.morphdbase.de/?J_vonDoehren_20160811-M-31.1](http://www.morphdbase.de/?J_vonDoehren_20160811-M-31.1) |
| **Fig 4C:** 2-*dpf*, Rfa-lir | FMRF/AF488 | (488) 30 | --- | (488) 580 | --- | (488) -0.4 | --- | (488)  495-560 | --- | 158 | 4-67 (64/69) | 1 | no | 55 | 37; -35 | 0.96 | 2-88 | [www.morphdbase.de/?J_vonDoehren_20160811-M-32.1](http://www.morphdbase.de/?J_vonDoehren_20160811-M-32.1) |
| **Fig 4D:** 3-*dpf*, Rfa-lir | FMRF/AF488 (tub/AF633) | (488) 34 | --- | (488) 600 | --- | (488) -0.4 | --- | (488)  495-560 | --- | 179 | 17-60 (44/63) | 1 | no | -43 | -20; 5 | 1 | 2-160 | [www.morphdbase.de/?J_vonDoehren_20160811-M-33.1](http://www.morphdbase.de/?J_vonDoehren_20160811-M-33.1) |
| **Fig 6A:** 4-*dpf* Rfa-lir | FMRF/AF488 (tub/AF633) | (488) 34 | --- | (488) 580 | --- | (488) -0.4 | --- | (488)  492-557 | --- | 168 | 18-61 (44/66) | 1 | yes | 172 | -25; -15 | 0.93 | 3-194 | [www.morphdbase.de/?J_vonDoehren_20160811-M-34.1](http://www.morphdbase.de/?J_vonDoehren_20160811-M-34.1) |
| **Fig 6B:** 5-*dpf*, Rfa-lir | FMRF/AF488 (tub/AF633) | (488) 34 | --- | (488) 550 | --- | (488) -0.4 | --- | (488)  495-560 | --- | 179 | 8-55 (48/60) | 1 | no | 170 | 40; -10 | 0.87 | 3-193 | [www.morphdbase.de/?J_vonDoehren_20160811-M-35.1](http://www.morphdbase.de/?J_vonDoehren_20160811-M-35.1) |
| **Fig 6C**: 7-*dpf*, Rfa-lir | FMRF/AF488 (tub/AF633) | (488) 34 | --- | (488) 570 | --- | (488) -0.4 | --- | (488)  495-560 | --- | 185 | 4-51 (48/55) | 1 | yes | 85 | 40; 0 | 1 | 4-160 | [www.morphdbase.de/?J_vonDoehren_20160811-M-36.1](http://www.morphdbase.de/?J_vonDoehren_20160811-M-36.1) |
| **Fig 6D:** 10-*dpf*, Rfa-lir | FMRF/AF488 (tub/AF633) | (488) 34 | --- | (488) 570 | --- | (488) -0.4 | --- | (488)  495-560 | --- | 192 | 7-46 (40/50) | 1 | yes | -162 | 5; -15 | 1 | 2-125 | [www.morphdbase.de/?J_vonDoehren_20160811-M-37.1](http://www.morphdbase.de/?J_vonDoehren_20160811-M-37.1) |

^1^) AF – AlexaFluor, FMRF – antibody against FMRF-amides, syn – antibody against SYNORF1 (3C11), tub – antibody against acetylated α-tubulin, 5HT – antibody against serotonin.

^2^) manual cleaning refers to evident fluorescent particles outside of the specimens (see e.g. Fig S1 A & D) that were digitally dimmed under optical control.
